# Community science data suggests that urbanization and forest habitat loss threaten aphidophagous native lady beetles

**DOI:** 10.1002/ece3.7229

**Published:** 2021-02-21

**Authors:** Mary M. Gardiner, Kayla I. Perry, Christopher B. Riley, Katherine J. Turo, Yvan A. Delgado de la flor, Frances S. Sivakoff

**Affiliations:** ^1^ Department of Entomology The Ohio State University Columbus OH USA; ^2^ Bartlett Tree Research Laboratories Charlotte NC USA; ^3^ California Department of Pesticide Regulation Sacramento CA USA; ^4^ Department of Evolution, Ecology and Organismal Biology The Ohio State University Marion OH USA

**Keywords:** alien, citizen science, Coccinellidae, exotic, exploitative competition, habitat compression, *Harmonia axyridis*, invasive, ladybird

## Abstract

1. Community scientists have illustrated rapid declines of several aphidophagous lady beetle (Coccinellidae) species. These declines coincide with the establishment of alien coccinellids. We established the Buckeye Lady Beetle Blitz program to measure the seasonal occupancy of coccinellids within gardens across a wide range of landscape contexts. Following the Habitat Compression Hypothesis, we predicted that gardens within agricultural landscapes would be alien‐dominated, whereas captures of natives would be higher within landscapes encompassing a high concentration of natural habitat.
2. Within the state of Ohio, USA, community scientists collected lady beetles for a 7‐day period across 4 years in June and August using yellow sticky card traps. All identifications were verified by professional scientists and beetles were classified by three traits: status (alien or native), mean body length, and primary diet. We compared the relative abundance and diversity of coccinellids seasonally and determined if the distribution of beetles by size, status, and diet was related to landscape features.
3. Alien species dominated the aphidophagous fauna. Native aphidophagous coccinellid abundance was positively correlated with forest habitat while alien species were more common when gardens were embedded within agricultural landscapes. Urbanization was negatively associated with both aphidophagous alien and native coccinellids.
4. *Synthesis and Applications:* Our census of native coccinellid species within residential gardens—a widespread and understudied habitat—was enabled by volunteers. These data will serve as an important baseline to track future changes within coccinellid communities within this region. We found that native coccinellid species richness and native aphidophagous coccinellid abundance in gardens were positively associated with forest habitat at a landscape scale of 2 km. However, our understanding of when and why (overwintering, summer foraging, or both) forest habitats are important remains unclear. Our findings highlight the need to understand how declining aphidophagous native species utilize forest habitats as a conservation priority.

## Graphical abstract

We established the Buckeye Lady Beetle Blitz community program to track native lady beetle communities in residential gardens. We found a strong positive relationship between the abundance of forested habitat in the surrounding landscape and native aphidophagous coccinellid abundance.

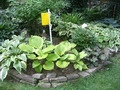

## 1 INTRODUCTION

Community science programs are pivotal tools for long‐term biodiversity monitoring (Toomey & Domroese, 2013). To date, such initiatives have detected and tracked invasive species (Brown et al., 2018), monitored habitat use by rare species (Campbell & Engelbrecht, 2018), and even rediscovered “extinct” taxa (Donnelly et al., 2014). Community scientists have undoubtedly contributed to improved habitat management as well through initiatives such as invasive species eradications (Tobin et al., 2014) and vector control (Johnson et al., 2018). Residential gardens are increasingly recognized as a potential refuge for native biodiversity (Goddard et al., 2010), but these private properties are difficult for scientists to access and study (Gaston et al., 2005; Goddard et al., 2010). Engaging community scientists enables biomonitoring efforts within residential gardens and facilitates the evaluation of conservation tactics promoted by wildlife gardening programs to support biodiversity. Further, as sampling can occur across many landscape contexts, community science programs are ideal to examine how landscape composition, configuration, and dynamics influence species distributions (Dickinson et al., 2012; Roy et al., 2016).

Given worldwide declines (Brown & Roy, 2018), aphidophagous lady beetles (Coleoptera, Coccinellidae) have become a major focus of community science monitoring programs (Gardiner et al., 2012; Grez et al., 2016; Losey et al., 2007; Roy & Brown, 2015). Lady beetles are charismatic species that capture public interest and provide a key ecosystem service by consuming aphids, scales, mildews, and other pests. Moreover, coccinellid communities have undergone a rapid species turnover, which has been tracked with volunteer biomonitoring efforts. Alien species are now the dominant aphidophagous coccinellid fauna across much of the world, whereas many native coccinellid species have declined (Alyokhin & Sewell, 2004; Bahlai et al., 2015; Roy, Brown, et al., 2016). For example, a community scientist was credited as “rediscovering” the aphidophagous nine‐spotted lady beetle *Coccinella novemnotata* Herbst in 2006; this once‐common beetle had not been collected in eastern North America in over a decade (Losey et al., 2007). In particular, the establishment and spread of the alien species *Coccinella septempunctata* L. and *Harmonia axyridis* (Pallis) coincide with the decline of several aphidophagous native coccinellid species (Gardiner et al., 2012; Losey et al., 2007; Roy, Brown, et al., 2016; Steffens & Lumen, 2015).

Both direct and indirect forms of competition with alien species have been proposed as mechanisms to explain the decline of aphidophagous native lady beetles (Pell et al., 2008). For example, intraguild predation of native coccinellid eggs and larvae by alien competitors has been repeatedly found under field conditions (Brown et al., 2015; Gagnon et al., 2011; Ortiz‐Martínez et al., 2020; Thomas et al., 2013). Evidence that native coccinellids are more likely to be the intraguild prey than intraguild predator (Katsanis et al., 2013; Snyder et al., 2004) suggests that these interactions may negatively influence native coccinellid populations. Likewise, evidence of apparent competition has been demonstrated, wherein alien species benefit from lower egg predation rates as compared to natives. For example, in its native range, *Hippodamia convergens* Guérin‐Méneville experienced greater egg predation than the alien *H. axyridis* from a guild of shared predators (Smith & Gardiner, 2013a, 2013b) potentially due to differences in concentrations of defensive alkaloids (Kajita et al., 2010).

The introduction of alien coccinellids has also increased the degree of exploitative competition experienced by native predators within aphidophagous guilds (Alyokhin & Sewell, 2004; Bahlai et al., 2015; Evans, 2000, 2004; Evans et al., 2011; Howe et al., 2016). The “Habitat Compression Hypothesis” suggests that exploitative competition from alien species for shared aphid prey has resulted in a “compression” of the foraging habitats some native lady beetles are able to exploit (Alyokhin & Sewell, 2004; Bahlai et al., 2015; Evans, 2000, 2004; Evans et al., 2011). This hypothesis predicts that native aphidophagous lady beetles are likely to occur at low frequencies in agricultural and urban habitats where alien species are superior competitors and will be more likely to persist in natural habitats where alien species are less common (Bahlai et al., 2015; Evans, 2004; Grez et al., 2013; Parker et al., 2020). Support for the Habitat Compression Hypothesis was found in central Chile, where the proportion of alien to native coccinellid individuals increased across a disturbance gradient that included agricultural and natural habitats (Grez et al., 2013). Likewise, long‐term data from Michigan, USA, demonstrated that habitat use patterns of the native coccinellid *Adalia bipunctata* L. shifted from agricultural habitats to forest patches following the introduction of alien competitors (Bahlai et al., 2015). At the landscape scale, the Habitat Compression Hypothesis predicts that natural habitats will serve as a source of native coccinellids to recolonize managed habitats when exploitative competition is relaxed (Evans, 2004). Positive relationships between the abundance of native coccinellids in croplands and the concentration of natural habitats in the landscape have been documented (Gardiner et al., 2009), lending support to this hypothesis. However, alien species have frequently been found to dominate the coccinellid fauna found in natural habitats (Finlayson et al., 2008; Gardiner et al., 2010; Smith & Gardiner, 2013a, 2013b) which questions if these landscape elements consistently act as refugia and sources of aphidophagous native coccinellids.

We developed a community science program to determine if the concentration of natural habitat in the landscape predicted the abundance of coccinellids in managed residential gardens, following the predictions of the Habitat Compression Hypothesis. The Buckeye Lady Beetle Blitz community science program quantified the relative abundance and species richness of native and alien lady beetles within residential gardens across Ohio, USA. These residential gardens were embedded within urban, suburban, and rural landscapes that varied in the proportion of natural habitat present. We predicted that alien species would be common within residential gardens (Prediction 1). However, following the Habitat Compression Hypothesis, we expected that natural landscape elements would serve as source habitats of native aphidophagous coccinellids. Therefore, we predicted that gardens within landscapes with a high proportion of natural habitat would be occupied by the greatest abundance and species richness of aphidophagous native lady beetles, whereas gardens embedded in agricultural and urban landscapes lacking sufficient refugia would be dominated by alien coccinellid species (Prediction 2).

## 2 MATERIALS AND METHODS

### 2.1 Buckeye Lady Beetle Blitz community science program

**FIGURE 1:**
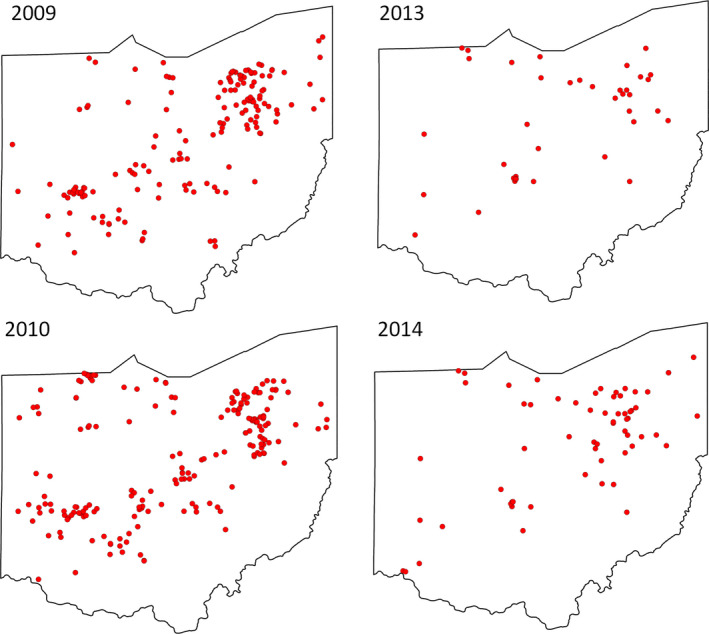
Locations of residential gardens sampled by Buckeye Lady Beetle Blitz community scientists in 2009 (*n* = 161), 2010 (*n* = 194), 2013 (*n* = 43), and 2014 (*n* = 64)

The Buckeye Lady Beetle Blitz was initiated in 2009 and data collection occurred in 2009, 2010, 2013, and 2014 throughout the state of Ohio, USA (Figure 1). The name of our program was inspired by Ohio being the “buckeye state” due to its state tree, the Ohio buckeye (*Aesculus glabra*), and “Brutus Buckeye”—The Ohio State University mascot. The majority of community scientists we engaged were members of The Ohio State University Extension (OSUE) Master Gardener Volunteer program (https://mastergardener.osu.edu), whose active community volunteers complete an intensive horticultural training program. The mission of the program is to empower trained volunteers to educate others with research‐based gardening information. The OSUE Master Gardener Volunteer program focuses on several initiatives including integrated pest management, invasive species detection and eradication/management, backyard and local foods, and environmental horticulture. To participate in the Buckeye Lady Beetle Blitz program, community scientists were required to attend an in‐person training workshop, held during the spring (March–May) and often in conjunction with other OSUE Master Gardener Volunteer events to draw a larger number of participants from around the state. The number and locations of workshops varied annually based on budgetary constraints and our ability to partner with other OSUE Master Gardener Volunteer events, although one workshop was held in Wooster and Cleveland, Ohio, USA, annually. The total number of workshops held per year varied from 2 to 11.

At our workshops, attendees received a toolkit that contained step‐by‐step instructions, data sheets, an identification guide, two yellow sticky card traps with twist ties, a step‐in fence post, and two pre‐paid mailing envelopes (S1). Our full training program lasted approximately three hours. First, a 45‐min presentation was delivered by Dr. Mary Gardiner that explained all data collection protocols. Next, a live demonstration of how to deploy, collect, and mail the yellow sticky card traps was provided by Dr. Gardiner's graduate students and staff. During the remaining portion of the workshop, community scientists received hands‐on experience with lady beetle identification, which included time spent studying labeled species with a hand lens and their identification guide, an identification quiz wherein volunteers identified unknown specimens, and additional time to review any specimens incorrectly identified during the quiz. Dr. Gardiner and her graduate students and staff answered all identification questions that arose during the hands‐on training and following the quiz. An instructional video was also posted to YouTube and linked to the project website, which allowed community scientists the ability to review all Buckeye Lady Beetle Blitz procedures.

### 2.2 Buckeye Lady Beetle Blitz data collection

Community scientists were asked to select a garden site either at their home or at another residence where they had permission to sample. These sites were not visited by researchers and could be of any size and include vegetable crops, ornamental flowers, or a mixture of both. We asked our community scientists to indicate the size of their garden and describe what was planted on their data sheet. We aimed to include two categorical predictors in our analysis based on these data: Garden Area (Length x Width) and Garden Type (Vegetable Garden, Ornamental Flower Garden, Vegetable + Ornamental Flower Garden) (S1B). However, few volunteers provided this information, and we were not able to include these predictors in further analyses. Therefore, this represents a limitation in our dataset as we were not able to measure local effects. Nonetheless, the major goal of our study was to examine how landscape features influenced occupancy of gardens by native aphidophagous coccinellids, which was feasible with the data provided by our community scientists.

Each year, community scientists collected lady beetle data from their garden for 7 days (Sunday–Saturday) in June and again in August. For each month, volunteers selected from two potential 7‐day periods listed on their sampling instruction card. To initiate data collection, a step‐in fence post was established in the center of the garden, and a yellow sticky card trap was attached to the post at a 0.94 m height. After 7 days, community scientists collected their trap and recorded the number of four alien species and 11 native species captured on their data sheet (S2). For each of the two sampling months, community scientists returned their completed data sheet and yellow sticky card trap to The Gardiner Laboratory in a prepaid mailing envelope. Beyond the 15 species tracked by community scientists, our research team also recorded the abundance of *Hyperaspis undulata* (Say) and *Mulsantina picta* (Randall). We did not ask our community scientists to track *H. undulata* due to its small size and similar coloration to other small beetles captured on yellow sticky card traps. With a habitat affinity for coniferous forests, we did not include *M. picta* in our identification guide but recorded one individual.

### 2.3 Lady beetle species verification and characterization

Lady beetle species identifications were verified using Gordon (1985) following submission of the yellow sticky traps and data sheets by community scientists. For volunteer accuracy rates see Gardiner et al. (2012). Lady beetle species were characterized based on three traits: mean body length (mm), status (native or alien to eastern North America), and primary diet (aphids, scales, or mildew fungi) (Dixon & Dixon, 2000; Gordon, 1985; Hodek et al., 2012; Majerus, 2016). Diet classifications for each species were based on the most frequently reported prey.

### 2.4 Landscape data – Geoprocessing and analysis

Landscape data for 2009, 2010, 2013, and 2014 were obtained from the USDA Cropland Data Layer (CDL), which is a crop‐specific raster data layer generated annually for the continental United States using 30 m resolution satellite imagery and ground truthing (USDA National Agricultural Statistics Service, 2020). All landscape geoprocessing was performed in ArcGIS 10.1 (ESRI, 2011), where landscape composition was measured at circular buffers with 200 m and 2 km radii. The 200 m radius landscape was selected to measure the effect of edge habitats (Egerer et al., 2017; Rand & Louda, 2006), which have been demonstrated to influence the strength of competitive interactions occurring within coccinellid guilds (Gardiner et al., 2011). The 2 km radius landscape scale was included to account for the capacity of lady beetles to make long dispersal flights (Jeffries et al., 2013) as this encompasses the dispersal range cited for many native aphidophagous species (Gordon, 1985). Further, when landscape buffer radii of 0.5–3 km were compared, Gardiner et al. (2009) demonstrated that landscape features at 2 km were most predictive of native coccinellid distributions.

The Tabulate Area 2 tool in the Spatial Analyst Supplemental Toolbox v1.3 was used to calculate the number of pixels of each land cover class occurring within the extent of each buffer in the layer. Only land cover classes that represented 0.5 percent or greater of total land cover at 2 km when averaged across sites and years were included. This resulted in eleven land cover classes that accounted for over 98% of all land cover: 1. Deciduous Forest (NLCD; 20.9%), 2. Developed/Low Intensity (NLCD; 19.0%), 3. Developed/Open Space (NLCD; 17.3%), 4. Grassland/Pasture (NLCD; 11.6%), 5. Soybeans (USDA NASS; 9.2%), 6. Developed/Medium Intensity (NLCD; 7.4%), 7. Corn (USDA NASS; 6.6%), 8. Developed/High Intensity (NLCD; 3.0%), 9. Open Water (NLCD; 1.5%), 10. Winter Wheat (USDA NASS; 1.1%), and 11. Alfalfa (USDA NASS; 0.6%). All remaining categories were aggregated into class 12 “Other” (NLCD and USDA NASS; 1.8%) (S3). Using these data, we calculated *Percentage Cropland* (Corn, Soybean, Winter Wheat, and Alfalfa), *Crop Richness* (number of crop classes occurring within a buffer), *Percentage Forest*, *Percentage Grassland*, *Percentage Urban Developed* (Developed/Low, Medium, and High Intensity), and *Percentage Urban Greenspace* (Developed/Open Space). Landscape diversity was measured using the *Shannon Diversity Index* (Peet, 1974).

### 2.5 Statistical analysis

To examine temporal variation in the abundances of native and alien aphidophagous lady beetles, we developed generalized linear models (GLMs) using the Poisson distribution. Generalized linear models are a commonly used method for analyzing count data within ecology (Warton et al., 2016). Each data collection year was analyzed independently to avoid issues of temporal autocorrelation as certain residential gardens were included in multiple years, depending on whether community scientists decided to participate repeatedly. For a given year, three models were developed, each incorporating one of three response variables: (a) *Native Aphidophagous* (abundance of native aphid‐feeding coccinellids that compete for prey with alien species), (b) *Native Other* (abundance of native coccinellids that do not feed primarily on aphids or compete directly with alien species for prey), and (c) *Alien* (abundance of introduced lady beetles, all species are aphidophagous). The categorical predictor used in each model was *Sampling Time* (June or August). Patterns were deemed significant when model coefficients had *p*‐values < 0.05. All GLMs were developed using the package “lme4” (Bates et al., 2015) in R version 3.6.0 (R Core Team, 2019).

Partial Least Squares Canonical Analysis (PLSCA) and relevance network analysis were used to evaluate the relationships between lady beetle assemblages and landscape factors. PLSCA analyzes the linear relationships between variables in two matrices by deriving a latent variable from each matrix to maximize the covariance explained between them (Abdi & Williams, 2013). Partial least squares methods are capable of analyzing multiple collinear response variables from data with small sample sizes relative to the number of dependent variables (Carrascal et al., 2009). PLSCA considers all variables dependent and compares them as a canonical correlation (i.e., variables are not identified as response or predictors a priori). We used a correlation coefficient threshold of 0.5 to establish associations among variables, with coefficients higher than 0.5 or lower than −0.5 on either axis considered significant. Variables with significant correlation coefficients were retained for the relevance network analysis, which calculates a pairwise similarity matrix. Similarity values approximate a Pearson correlation and are calculated by summing the correlations between individual variables and each of the latent variables from the PLSCA. A 0.5 threshold was used to evaluate the strength of variable associations, with similarity values higher than 0.5 or lower than −0.5 considered significant. PLSCA and relevance network analysis were performed using the package “mixOmics” (Rohart et al., 2017) in R.

Two PLSCA analyses were conducted, one for each landscape radius (200 m and 2 km). In each analysis, the following community metrics were included: *Native Shannon Diversity*, *Native Body Length*, *Alien Body Length*, *Total Native Abundance*, *Native Aphidophagous Abundance*, *Native Coccidophagous Abundance*, *Native Fungivorous Abundance*, and *Alien Abundance*. Each analysis also included the landscape variables *Shannon Diversity Index*, *Crop Richness*, *Percentage Cropland*, *Percentage Urban Developed*, *Percentage Urban Greenspace*, *Percentage Forest*, and *Percentage Grassland* at either the 200 m or 2 km radius surrounding each site. First, lady beetles collected from sticky traps deployed in June and August were pooled for each site. From these pooled data, we calculated the community metrics. The Shannon Diversity Index for native species was calculated using PC‐ORD software (McCune & Mefford, 2006). We calculated body length as the community‐weighed mean (cwm), which is the mean body length of lady beetles collected at a site weighted by the relative abundance of species. The cwm was calculated using the package “FD” (Laliberte et al., 2014) in R.

## 3 RESULTS

### 3.1 Lady beetle communities within residential gardens

**FIGURE 2:**
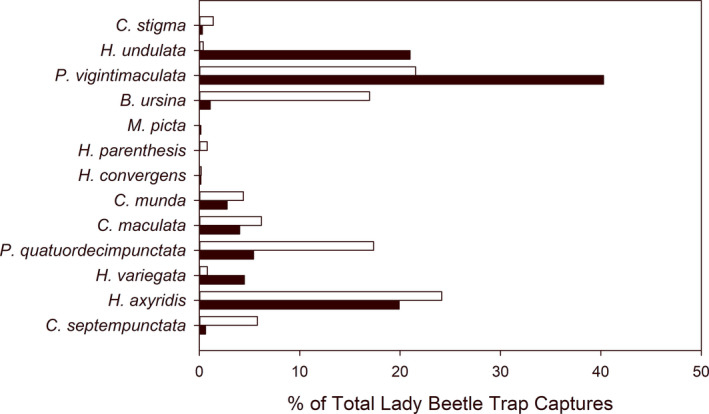
Contribution of each lady beetle species (% of total) to yellow sticky card trap catches in June and August. Percentages were calculated across the 4 years of data collection

A total of 1,136 lady beetles representing 13 species were collected via sticky traps from residential gardens (Table 1, Figure 2). *Psyllobora vigintimaculata* (Say), *H. axyridis*, and *H. undulata* were the most frequently captured, comprising 65.4% of the total lady beetles. Four alien species were found, *H. axyridis*, *Propylea quatuordecimpunctata* (L.), *C. septempunctata*, and *Hippodamia variegata* (Goeze), comprising 37.3% of the total number of lady beetles. Nine native species were captured, including two individuals of the native species *H. convergens* in 2009, which has been reported as in decline within the north central US (Gardiner et al., 2012; Steffens & Lumen, 2015). No individuals of *A. bipunctata*, *C. novemnotata*, *Coccinella trifasciata* L., or *Hippodamia tredecimpunctata* (L.) were captured. Native aphidophagous lady beetle abundance decreased from June to August in 2009, 2010, and 2014 (*p* < 0.05, S4). Alien coccinellids were more abundant in residential gardens in June than in August in 2009 and 2013 but increased across the growing season in 2010 (*p* < 0.05, S4). Native coccinellids that feed on scale insects or fungi increased across the growing season each year (*p* < 0.001, S4).

**TABLE 1:** Abundance and traits of native and alien lady beetle species collected from residential gardens by Buckeye Lady Beetle Blitz community scientists across the state of Ohio, USA

Subfamily	Tribe	Species	Total abundance	Status	Mean body length (mm)	Primary diet
Scymninae	Hyperaspidini	*Brachiacantha ursina* (Fabricius)	123	Native	3.5	Aphidoidea, Coccoidea
		*Hyperaspis* *undulata* (Say)	139	Native	2.3	Coccoidea
Chilocorinae	Chilocorini	*Chilocorus stigma* (Say)	14	Native	4.4	Coccoidae, Aphidoidea
Coccinellinae	Coccinellini	*Coccinella septempunctata* (Linnaeus)	38	Exotic	7.2	Aphidoidea
		*Coleomegilla maculata* (Degeer)	67	Native	5.4	Aphidoidea, Pollen
		*Cycloneda munda* (Say)	52	Native	4.7	Aphidoidea
		*Harmonia axyridis* (Pallas)	333	Exotic	6.5	Aphidoidea
		*Hippodamia convergens* Guerin	2	Native	5.8	Aphidoidea
		*Hippodamia parenthesis* (Say)	4	Native	4.7	Aphidoidea
		*Hippodamia variegata* (Goeze)	34	Exotic	4.5	Aphidoidea
		*Mulsantina picta* (Randall)	1	Native	4.3	Aphidoidea
		*Propylea quatuordecimpunctata* (Linnaeus)	162	Exotic	4.4	Aphidoidea
	Psylloborini	*Psyllobora vigintimaculata* (Say)	501	Native	2.4	Fungi (Erisyphaceae)

### 3.2 Landscape drivers of lady beetle distributions

**FIGURE 3:**
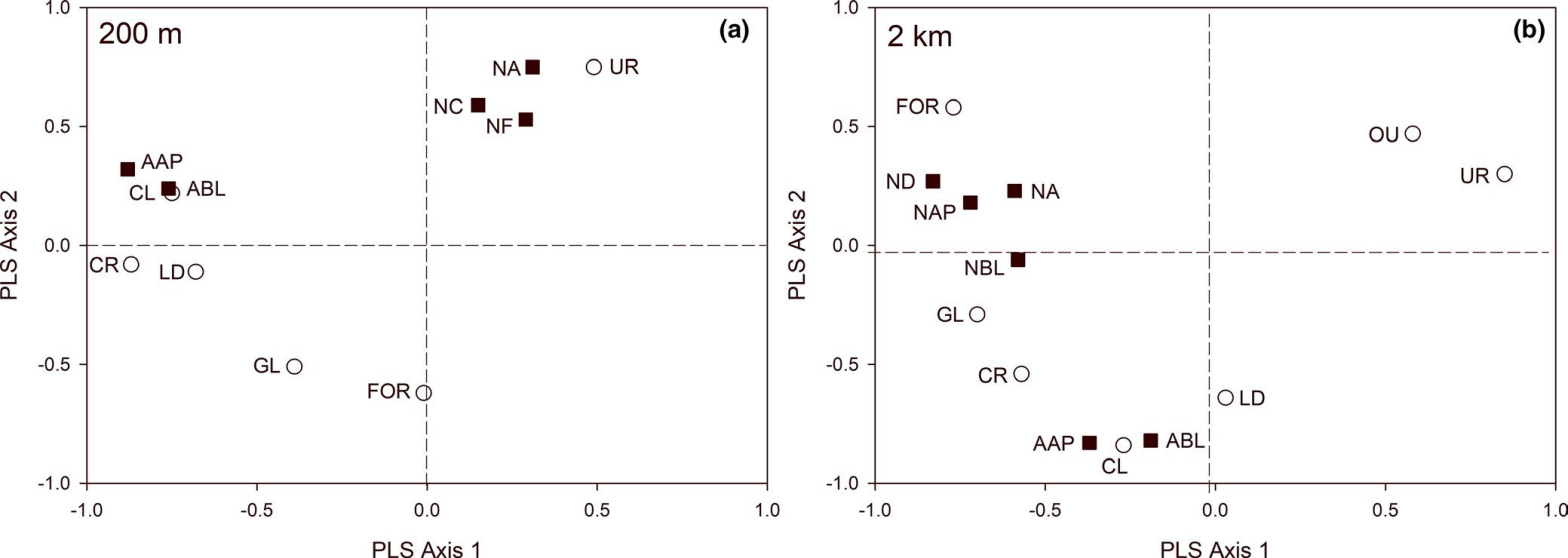
Partial Least Squares Canonical Analysis (PLSCA) plot for lady beetle community metrics and landscape features at 200 m (a) and 2 km (b). Total variance explained by axes 1 and 2 at 200 m was 54.2% and 18.3%, respectively. Total variance explained at 2 km was 67.7% for axis 1 and 53.1% for axis 2. Variables with correlation coefficients higher than 0.5 or lower than −0.5 on either axis are shown. The strength and direction of relationships in PLSCA are determined by relative distance, with closer variables being positively correlated to one another. *Lady beetle assemblage metrics* are squares and labeled as follows: Total Native Abundance (NA), Native Coccidophagous Abundance (NC), Native Fungivorous Abundance (NF), Native Shannon Diversity (ND), Native Body Length (NBL), Alien Body Length (ABL), Native Aphidophagous Abundance (NAP), and Alien Abundance (AAP). *Landscape features* are circles and labeled as follows: Percentage Urban Developed (UR), Percentage Urban Greenspace (OU), Percentage Cropland (CL), Crop Richness (CR), Percentage Grassland (GL), Percentage Forest (FOR), Shannon Diversity Index (LD)

**FIGURE 4:**
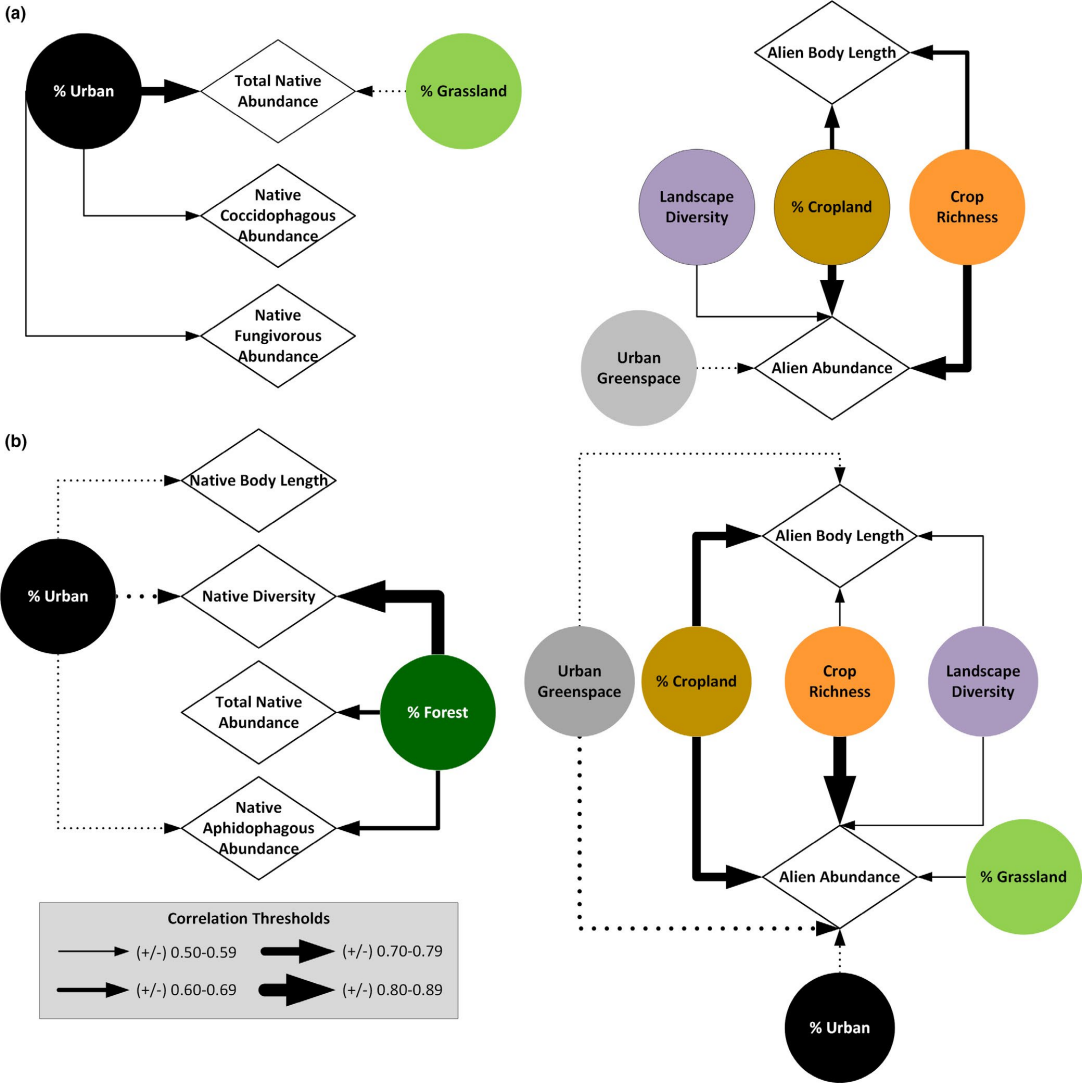
Relevance network plot for lady beetle metrics (Native Shannon Diversity, Native Body Length, Alien Body Length, Total Native Abundance, Native Aphidophagous Abundance, Native Coccidophagous Abundance, Native Fungivorous Abundance, and Alien Abundance) and surrounding landscape variables (Shannon Diversity Index, Crop Richness, Percentage Cropland, Percentage Urban Developed, Percentage Urban Greenspace, Percentage Forest, and Percentage Grassland) at 200 m (a) and 2 km (b). Variables with PLSCA correlation coefficients higher than 0.5 or lower than −0.5 on either axis were retained for the relevance network analysis. Solid lines indicate positive associations, whereas dashed lines are negative associations. Thickness of the lines indicate the strength of the association between the two variables, with thicker lines having a stronger similarity value. Relevance network similarity values for lady beetle community metrics are provided in Table 3

Distinct landscape variables predicted native versus alien lady beetle communities within gardens (Figures 3, 4, Tables 2, 3). At 200 m, *Total Native Abundance, Native Coccidophagous Abundance*, and *Native Fungivorous Abundance* were positively associated with *Percentage Urban Developed,* and *Total Native Abundance* was negatively related to *Percentage Grassland*, as indicated by the pairwise similarity values from the relevance network analysis (Figures 3a, 4a, Table 3). At 200 m, *Alien Abundance* was positively correlated with *Percentage Cropland, Cropland Richness,* and *Landscape Diversity,* and negatively related to *Percentage Urban Greenspace* (Figures 3a, 4a, Table 3). *Alien Body Length* also was positively correlated with *Percentage Cropland* and *Cropland Richness* at the 200 m landscape scale (Figures 3a, 4a, Table 3).

**TABLE 2:** Partial Least Squares Canonical Analysis (PLSCA) correlation coefficients for lady beetle diversity and traits and landscape features at 200 m and 2 km

Variables	200 m		2 km	
	Axis 1 (t1)	Axis 2 (t2)	Axis 1 (t1)	Axis 2 (t2)
Lady beetle species				
Total native abundance (NA)	0.31	0.75	−0.59	0.23
Native Shannon diversity (ND)	−0.03	−0.17	−0.83	0.27
Native body length (NBL)	−0.04	0.34	−0.58	−0.06
Native aphidophagous abundance (NAP)	−0.01	−0.09	−0.72	0.18
Native coccidophagous abundance (NC)	0.15	0.59	−0.35	0.21
Native fungivorous abundance (NF)	0.29	0.53	−0.33	0.08
Alien abundance (AAP)	−0.88	0.32	−0.37	−0.83
Alien body length (ABL)	−0.76	0.24	−0.19	−0.82
Landscape variables				
Shannon diversity (LD)	−0.68	−0.11	0.03	−0.64
Crop richness (CR)	−0.87	−0.08	−0.57	−0.54
Percentage cropland (CL)	−0.75	0.22	−0.27	−0.84
Percentage urban developed (UR)	0.49	0.75	0.85	0.30
Percentage urban greenspace (OU)	0.46	−0.27	0.58	0.47
Percentage forest (FOR)	−0.01	−0.62	−0.77	0.58
Percentage grassland (GL)	−0.39	−0.51	−0.70	−0.29

Note

Only variables with a correlation coefficient higher than 0.50 or lower than −0.50 on either axis were considered significant. Total variance explained by axes 1 and 2 at 200 m was 54.2% and 18.3%, respectively. Total variance explained at 2 km was 67.7% for axis 1 and 53.1% for axis 2.

**TABLE 3:** Pairwise similarity matrix calculated from relevance association network analysis following the Partial Least Squares Canonical Analysis (PLSCA) for lady beetle diversity and traits and landscape variables at 200 m and 2 km

Landscape variables	NA		ND		NBL		NAP		NC		NF		ABL		AAP	
	200 m	2 km	200 m	2 km	200 m	2 km	200 m	2 km	200 m	2 km	200 m	2 km	200 m	2 km	200 m	2 km
Shannon diversity														0.52	0.56	0.52
Crop richness													0.64	0.56	0.74	0.67
Percentage cropland													0.62	0.75	0.73	0.81
Percentage urban developed	0.72			−0.62		−0.51		−0.56	0.52		0.55					−0.57
Percentage urban greenspace														−0.50	−0.50	−0.61
Percentage forest		0.60		0.80				0.67								
Percentage grassland	−0.51															0.51

Note

The similarity values are calculated by summing the correlations between the individual variables and each of the latent components from the PLSCA model. These similarity values approximate a Pearson correlation coefficient. A 0.5 threshold value was used for the relevance network analysis, and associations that met this threshold are shown. Lady beetle assemblage metrics are listed as follows: Total Native Abundance (NA), Native Shannon Diversity (ND), Native Body Length (NBL), Alien Body Length (ABL), Native Aphidophagous Abundance (NAP), Alien Abundance (AAP), Native Coccidophagous Abundance (NC), and Native Fungivorous Abundance (NF).

At 2 km, *Native Shannon Diversity*, *Native Aphidophagous Abundance,* and *Native Body Length* were negatively associated with *Percentage Urban Developed* (Figures 3b, 4b, Table 3). *Total Native Abundance*, *Native Aphidophagous Abundance*, and *Native Shannon Diversity* were positively related to *Percentage Forest* at the 2 km landscape scale (Figures 3b, 4b, Table 3). *Alien Abundance* and *Alien Body Length* were positively associated with *Landscape Diversity*, *Cropland Richness*, and *Percentage Cropland*, and negatively associated with *Percentage Urban Greenspace* (Figures 3b, 4b, Table 3). *Alien Abundance* was also positively associated with *Percentage Grassland* and negatively associated with *Percentage Urban Developed* (Figures 3b, 4b, Table 3).

## 4 DISCUSSION

Community science programs across three continents have documented rapid changes in coccinellid community composition (Grez et al., 2016; Losey et al., 2007; Roy et al., 2012). Community science volunteers have quantified the severity and geographic extent of native lady beetle declines (Gardiner et al., 2012; Losey et al., 2007; Roy et al., 2012), such as 30% and 44% reductions in *A. bipunctata* abundance over 5 years across Belgium and Great Britain, respectively (Roy et al., 2012). Community scientists have also tracked the rapid spread of alien coccinellids such as *H. axyridis* across central Chile (Grez et al., 2016), the UK (Roy & Brown, 2015), and the United States (Gardiner et al., 2012). Building on this legacy, Buckeye Lady Beetle Blitz community scientists documented four alien species and nine native coccinellids within residential gardens in Ohio, USA, including two captures of *H. convergens*, which is now regionally rare (Gardiner et al., 2012; Steffens & Lumen, 2015). By networking across rural, suburban, and urban landscapes, Buckeye Lady Beetle Blitz volunteers also revealed that forest habitat is a strong positive driver of remaining native aphidophagous coccinellid populations, whereas, urbanization was negatively associated with the abundance of these species.

Historically, the geographic distributions of *A. bipunctata*, *H. convergens*, *C. novemnotata*, *C. transversogutta*, and *H. tredecimpunctata* extended across our study region (Gordon, 1985). Following early records (Bubna, 1902; Dury, 1879), these aphidophagous species were documented within the region for the next century (Dailey et al., 1978; Guyton, 1924; Holdsworth, 1970; Walker et al., 1984; Williams et al., 1995). For example, *A. bipunctata, C. transversoguttata*, and *H. convergens* were among the most abundant species found in a 1976 survey of common milkweed (*Asclepias syriaca* L.) growing within a railroad right‐of‐way habitat (Dailey et al., 1978; Gardiner et al., 2018). However, Buckeye Lady Beetle Blitz community scientists documented that dramatic changes in lady beetle species composition have occurred within Ohio. Volunteers failed to capture *A. bipunctata*, *C. novemnotata*, *C. trifasciata*, or *H. tredecimpunctata*, echoing the Lost Lady Bug community science project (Losey et al., 2012) which has infrequently observed these species in the Midwestern United States, and in some cases nationally.

Following our prediction, Buckeye Lady Beetle Blitz volunteers found that alien coccinellids dominated the predatory lady beetle fauna of residential gardens, with *H. axyridis* and *P. quatuordecimpunctata* being the most commonly collected species. The most abundant aphidophagous native species captured were *B. ursina*, *C. maculata,* and *C. munda*. This composition of alien and native species reflects contemporary communities collected from croplands and urban greenspaces in the region (Gardiner et al., 2012, 2014; Parker et al., 2020; Smith & Gardiner, 2013a). For example, in urban vacant lots, Parker et al. (2020) found that alien species dominated the coccinellid fauna representing 74% of captured specimens, and *B. ursina*, *C. maculata*, and *C. munda* were the most common aphidophagous native species collected from these sites. Nonetheless, even the most abundant aphidophagous native taxa captured by our community science survey could be experiencing population decline. For instance, regional reports of declines in *B. ursina* have been reported (Colunga‐Garcia & Gage, 1998). The changes in lady beetle composition documented by the Buckeye Lady Beetle Blitz survey underscore the importance of community science in capturing baseline data and supporting long‐term monitoring. The data provided by our volunteers will prove invaluable to track further population changes and measure the effectiveness of conservation strategies.

The Habitat Compression Hypothesis predicts that native aphidophagous coccinellids will occur at low frequencies in agricultural habitats, where alien species are superior competitors, and persist in natural habitats where competition from alien species is relaxed (Alyokhin & Sewell, 2004; Bahlai et al., 2015; Evans, 2000, 2004; Evans et al., 2011). Therefore, we predicted that native aphidophagous lady beetles would be more common in residential gardens as the concentration of natural habitat in the landscape increased. Our findings supported this prediction, as native aphidophagous coccinellid abundance and species richness were positively associated with the concentration of forest habitat present within a 2 km radius of residential gardens monitored by community scientists. Further supporting the Habitat Compression Hypothesis, the concentration of agricultural habitat as well as cropland diversity were positively associated with the abundance of alien species in residential gardens. Importantly, our survey results do not demonstrate why forest habitat was a positive driver of aphidophagous native coccinellid abundance. For instance, the Habitat Compression Hypothesis proposes that natural habitat acts as a refugia for native aphidophagous coccinellids escaping high exploitative competition during their breeding season (Evans, 2004). Alternatively, the positive association among native aphidophagous coccinellids and forest habitat may illustrate the dependence of these species on forests as overwintering habitat (Benton & Crump, 1979). Certainly, coccinellids are capable of long‐distance flights to access overwintering grounds (Hodek et al., 1993; Lee, 1980), but precise data on dispersal capacity is lacking for many species, and suitable overwintering habitat may be unavailable even at large spatial scales within some urban and suburban areas. For instance, the proportion of forested habitat within a 2 km radius landscape buffer varied widely across our study sites, from 1% to 78% of total land cover.

We found that native and alien aphidophagous coccinellids declined in residential gardens with increased urbanization. Our findings align with those from central Chile, where urbanization intensity was a negative driver of aphidophagous native abundance, native richness, and coccinellid body size within parks across a rural to urban landscape gradient (Grez et al., 2019). Likewise, increased impervious surface was negatively associated with lady beetle species richness within urban gardens in Berkshire, England (Rocha et al., 2018), and native and alien lady beetle abundance within vacant lots in Cleveland, Ohio (Parker et al., 2020). In contrast to aphidophagous species, Buckeye Lady Beetle Blitz volunteers found positive relationships between the density of the built environment and the abundance of coccidophagous and fungivorous lady beetles within their gardens. Positive associations between coccidophagous lady beetles and urbanization may be due to a greater resource abundance in cities. For example, arboreal scale populations are known to increase with elevated tree canopy temperatures resulting from increased impervious surfaces (Dale & Frank, 2014). Further, coccidophagous lady beetle species have been recorded to overwinter within leaf litter at the base of trees where they spend the breeding season foraging (Mayer & Allen, 1982). Therefore, these species would not need to navigate fragmented urban landscapes in autumn to seek a distinct overwintering habitat. Likewise, *P. vigintimaculata* feeds on powdery mildew fungi (Ascomycota: Erysiphales) (Sutherland & Parrella, 2009), and its high abundance within residential gardens and a positive association with urbanization may reflect greater resource availability within urban areas. Cities are known to have a higher plant richness than surrounding rural landscapes (Wania et al., 2006) and many herbaceous and woody ornamental plants as well as cultivated crops are susceptible to powdery mildew.

## 5 CONCLUSIONS

The Buckeye Lady Beetle Blitz program enabled the first regional assessment of lady beetle relative abundance in residential gardens in the United States. This effort provided a valuable dataset highlighting the severity of native aphidophagous lady beetle decline in the Midwestern United States and will serve as a baseline for future assessments. Our community science program demonstrated that a positive correlation exists between aphidophagous lady beetle abundance and forest habitats, which supports the Habitat Compression Hypothesis. However, this hypothesis implies that natural habitats act as refugia for aphidophagous native coccinellids from high exploitative competition during their breeding season. Further study is needed to determine if aphidophagous native species experience reduced exploitative competition by accessing forest habitats and if this aids their population stability. Alternative hypotheses, such as a dependence on forest cover for overwintering are possible and should be investigated. Further, conservation of native coccinellids may not simply rely on a threshold concentration of forest habitats within the landscape but also factors such as patch size, shape, quality, isolation, and distribution, which must be examined. The potential of urbanization as a negative driver of aphidophagous native lady beetle decline also warrants investigation. Urban land use increased by 36% from 1930 to 2010 across Ohio and surrounding states, whereas, agricultural habitat declined (Tayyebi et al., 2015), and the potential of these landscape dynamics to have influenced coccinellid compositional change should be studied. To devise a widespread conservation effort in our region, understanding how landscape composition, configuration, and dynamics might aid or hinder the maintenance of native aphidophagous coccinellid populations remains a critical next step.

## CONFLICT OF INTEREST

None.

## AUTHOR CONTRIBUTIONS

**Mary M. Gardiner:** Conceptualization (lead); funding acquisition (lead); investigation (lead); methodology (lead); project administration (lead); resources (lead); supervision (lead); visualization (lead); writing – original draft (lead); writing – review and editing (lead). **Kayla I. Perry:** Data curation (lead); formal analysis (lead); methodology (supporting); writing – review and editing (supporting)– Writing Original Draft (supporting). **Christopher B. Riley:** Data curation (supporting); formal analysis (supporting); methodology (supporting); writing – original draft (supporting); writing – review and editing (supporting). **Katherine J. Turo:** Writing‐review and editing (supporting)– data curation (supporting) – formal analysis (supporting). **Yvan A. Delgado de la flor:** Data curation (supporting); formal analysis (supporting); writing – review and editing (supporting). **Frances S. Sivakoff:** Formal analysis (supporting)– review editing (supporting).

## Supporting information

Supplementary Material

## Data Availability

Data has been deposited with the Dryad Digital Repository: https://doi.org/10.5061/dryad.cc2fqz657.
