# Supplementary material for: Community science data suggests that urbanization and forest habitat loss threaten aphidophagous native lady beetles
Source: Ecol Evol. 2021 Feb 21;11(6):2761–74. doi: 10.1002/ece3.7229 (PMC7981222; doi:10.1002/ece3.7229)
Supplement: Supplementary file 1 — Supplementary Material [file ECE3-11-2761-s001.docx]

**S1.** Community scientists who participated in the Buckeye Lady Beetle Blitz program received a toolkit that contained **(A)** step-by-step instructions, **(B)** data sheets, **(C)** a lady beetle identification guide, two yellow sticky card traps with twist ties, a step-in fence post, and two pre-paid mailing envelopes.

**A.** BLBB Sampling Instructions.

**
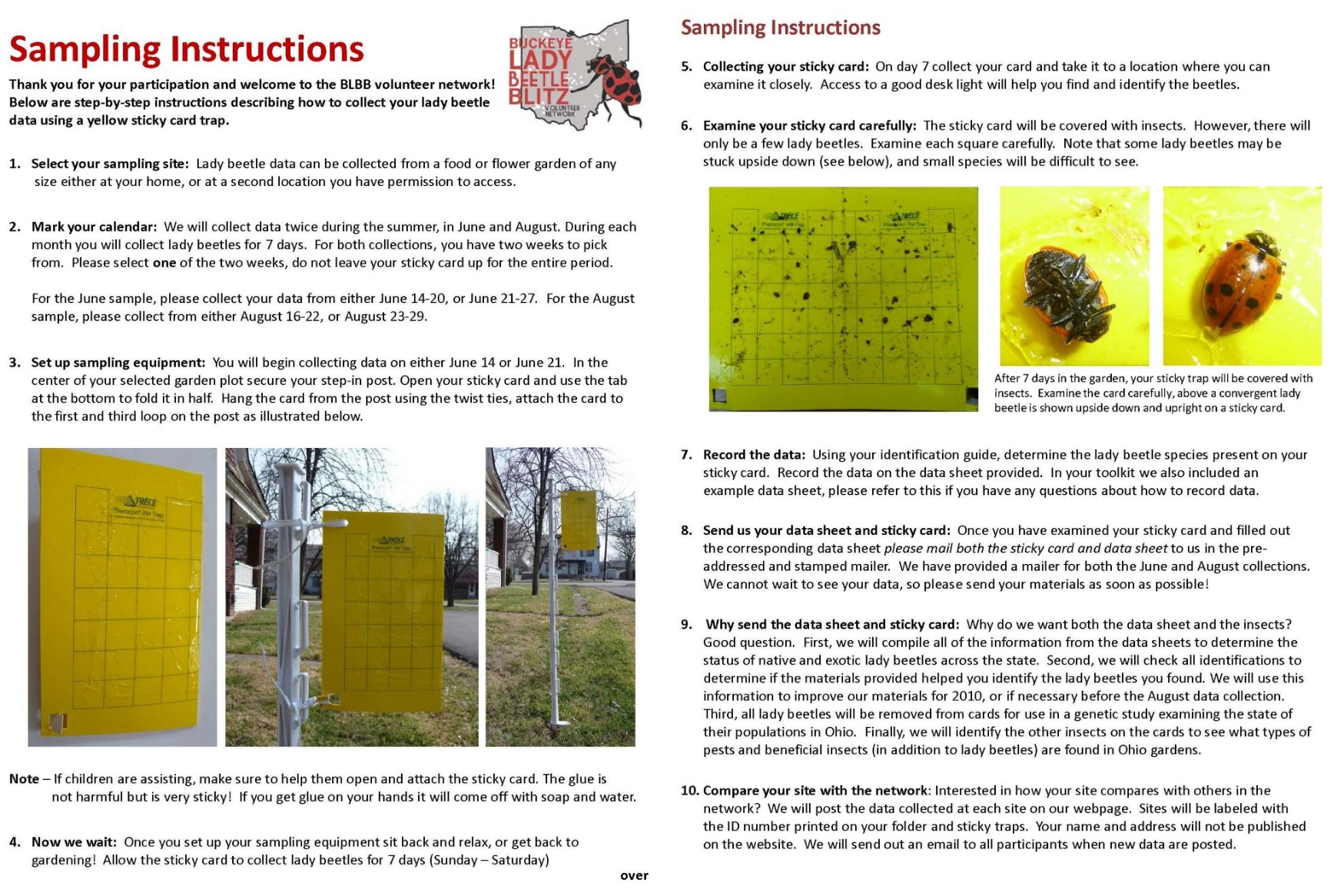
**

**B.** Volunteers received data sheets for both June and August. A blank June data sheet is shown here.

**
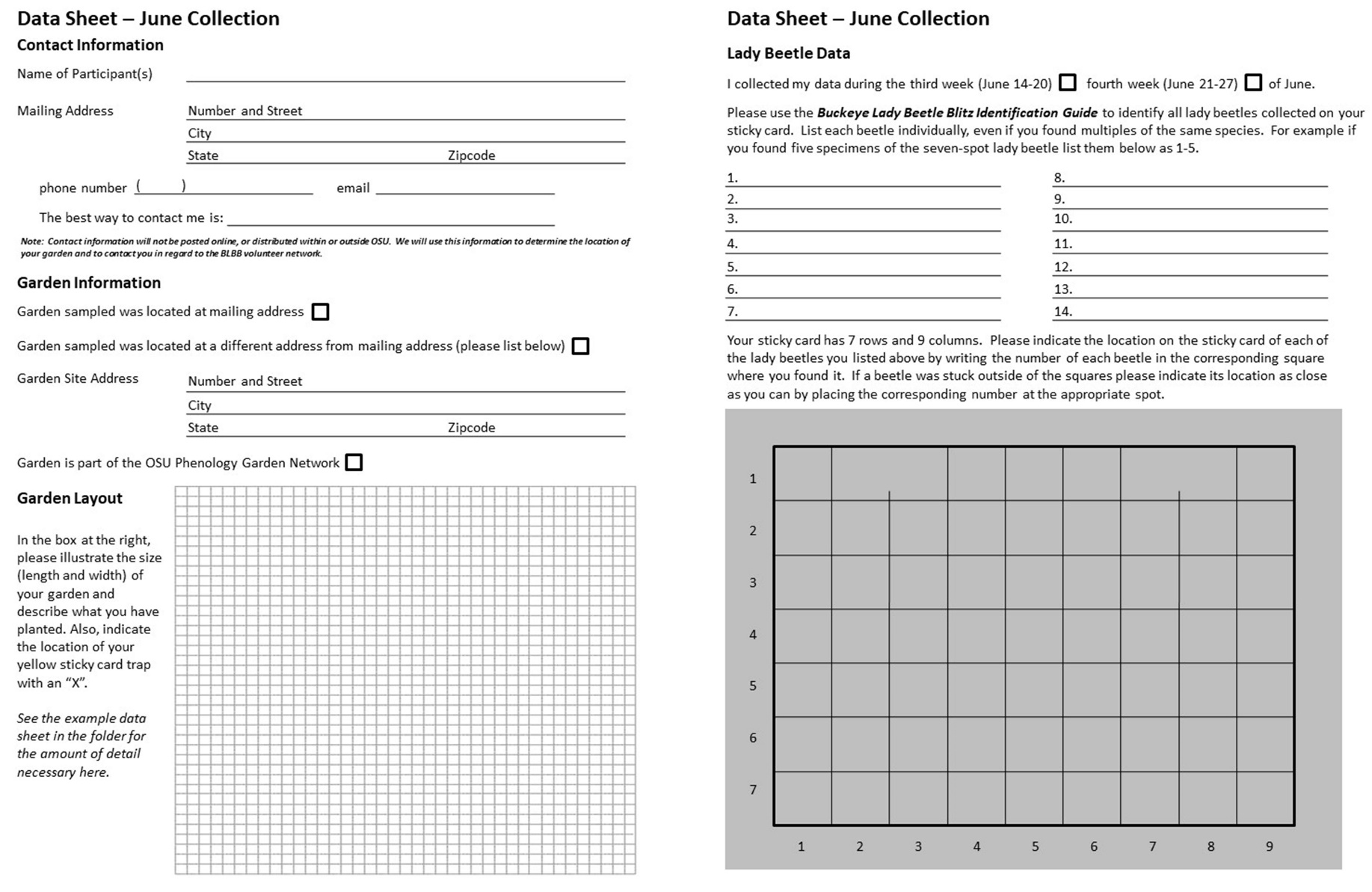
**

**C.** Four alien and 10 native species were included in the lady beetle guide provided to community scientists. We added a bookmark with identification information for an additional species, *Psyllobora vigintimaculata*, in 2010 due to its abundance on 2009 yellow sticky card traps. Note that this species was counted by our research team in 2009. Beyond these 15 species, our research team also recorded the abundance of *Hyperaspis undulata* and *Mulsantina picta*.

**
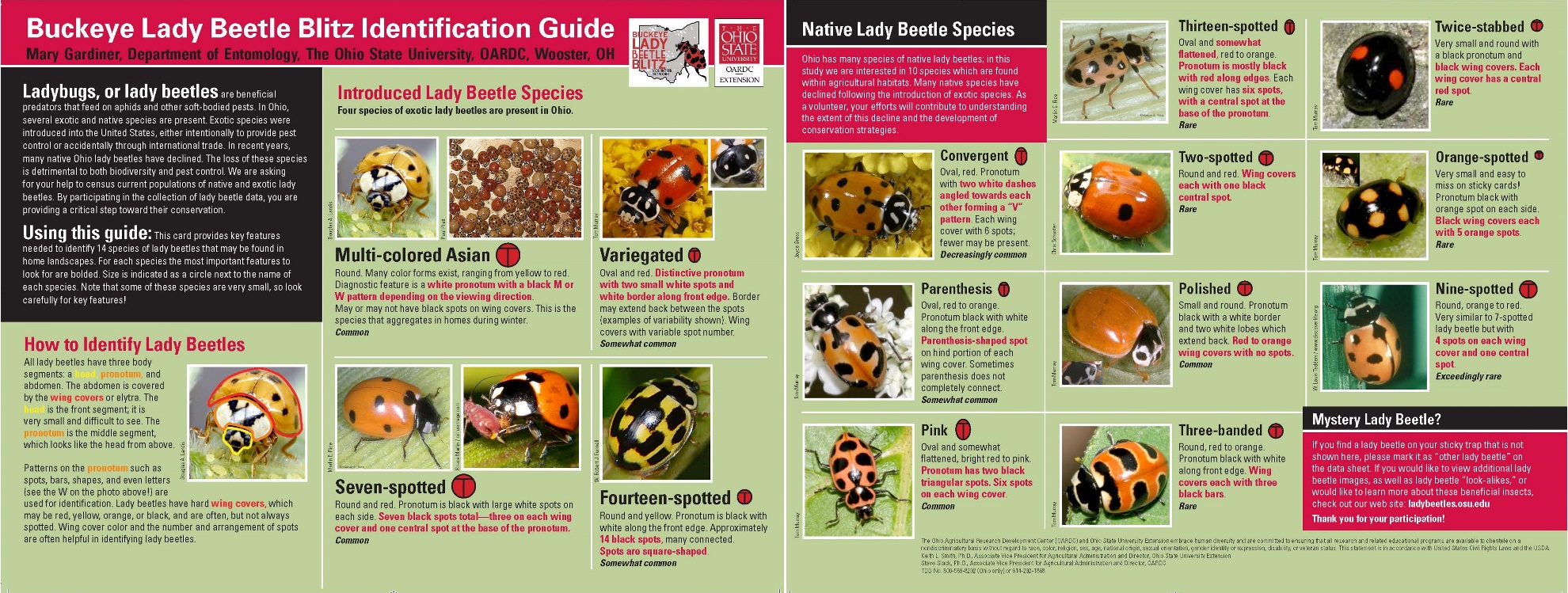
**

**S2.** Scientific and common names of lady beetle species tracked by BLBB volunteers.

| **Lady Beetle Species** | **Common Name Utilized in BLBB Identification Guide** |
| --- | --- |
| Exotic |  |
| *Coccinella septempunctata* | Seven-Spotted Lady Beetle |
| *Harmonia axyridis* | Multi-colored Asian Lady Beetle |
| *Hippodamia variegata* | Variegated Lady Beetle |
| *Propylea quatuordecimpunctata* | Fourteen-Spotted Lady Beetle |
|  |  |
| Native |  |
| *Adalia bipunctata* | Two-Spotted Lady Beetle |
| *Brachiacantha ursina* | Orange-Spotted Lady Beetle |
| *Chilocorus stigma* | Twice-Stabbed Lady Beetle |
| *Coccinella novemnotata* | Nine-Spotted Lady Beetle |
| *Coccinella transversoguttata* | Three-Banded Lady Beetle |
| *Coleomegilla maculata* | Pink Lady Beetle |
| *Cycloneda munda* | Polished Lady Beetle |
| *Hippodamia convergens* | Convergent Lady Beetle |
| *Hippodamia parantheses* | Parenthesis Lady Beetle |
| *Hippodamia tredecimpunctata* | Thirteen-Spotted Lady Beetle |
| *Psyllobora vigintimaculata* | Mildew Eating Lady Beetle |

**S3**. Complete list of land cover classes included in the 'Other' category. Note that for these classes to be included in 'Other', they had to account for less than 0.5% of total land cover within the 2 km radius buffers when averaged across study sites and years. Class names come directly from the USDA Cropland Data Layer.

| **Habitats Included in “Other” Category** |
| --- |
| Apple |
| Background |
| Barley |
| Barren |
| Cabbage |
| Carrots |
| Christmas Trees |
| Clover/Wildflowers |
| Cranberries |
| Cucumbers |
| Double Crop: Barley/Soybean |
| Double Crop: Corn/Soybean |
| Double Crop: Winter Wheat/Soybean |
| Double Crop: Winter Wheat/Corn |
| Double Crop: Winter Wheat/Sorghum |
| Dry Beans |
| Fallow/Idle Cropland |
| Flaxseed |
| Greens |
| Herbaceous Wetlands |
| Oats |
| Other Crops |
| Peaches |
| Peas |
| Peppers |
| Por or Orn Corn |
| Potatoes |
| Pumpkins |
| Rye |
| Shrubland |
| Sod/Grass Seed |
| Sorghum |
| Speltz |
| Squash |
| Strawberries |
| Sunflower |
| Sweet Corn |
| Tobacco |
| Tomatoes |
| Triticale |
| Woody Wetlands |

**S4.** Generalized linear mixed model results illustrating the direction and significance of lady beetle population abundance changes by group and across the growing season (June to August). Shaded cells indicate an increase in lady beetle abundance over time for a given group and year while white cells indicate a decrease. (***) = P < 0.001; (**) = P < 0.01; (*) = P < 0.05; NS = Not Significant.

|  | **Native Aphidophagous** | **Native Other** | **Alien** |
| --- | --- | --- | --- |
| *Year* |  |  |  |
| 2009 | **(***)** | **(***)** | **(***)** |
| 2010 | **(*)** | **(***)** | **(*)** |
| 2013 | **NS** | **(***)** | **(*)** |
| 2014 | **(***)** | **(***)** | **NS** |
